# Computer vision for microscopy diagnosis of malaria

**DOI:** 10.1186/1475-2875-8-153

**Published:** 2009-07-13

**Authors:** F Boray Tek, Andrew G Dempster, Izzet Kale

**Affiliations:** 1 Applied DSP & VLSI Research Group, University of Westminster, London, UK; 2 School of Surveying & Spatial Information Systems, University of New South Wales, Sydney, Australia

## Abstract

This paper reviews computer vision and image analysis studies aiming at automated diagnosis or screening of malaria infection in microscope images of thin blood film smears. Existing works interpret the diagnosis problem differently or propose partial solutions to the problem. A critique of these works is furnished. In addition, a general pattern recognition framework to perform diagnosis, which includes image acquisition, pre-processing, segmentation, and pattern classification components, is described. The open problems are addressed and a perspective of the future work for realization of automated microscopy diagnosis of malaria is provided.

## Background

Malaria is a serious infectious disease caused by a peripheral blood parasite of the genus *Plasmodium*. According to the World Health Organization (WHO), it causes more than 1 million deaths arising from approximately 300–500 million infections every year [1]. Although there are newer techniques [2], manual microscopy for the examination of blood smears [3] (invented in the late 19th century), is currently "the gold standard" for malaria diagnosis. Diagnosis using a microscope requires special training and considerable expertise [4]. It has been shown in several field studies that manual microscopy is not a reliable screening method when performed by non-experts due to lack of training especially in the rural areas where malaria is endemic [5-7]. An automated system aims at performing this task without human intervention and to provide an objective, reliable, and efficient tool to do so.

An automated diagnosis system can be designed by understanding the diagnostic expertise and representing it by specifically tailored image processing, analysis and pattern recognition algorithms. Although it is not a popular research topic, a noticeable number of vision studies directly address the automated diagnosis of malaria [8-16]. Despite being very specialized, if the fatality figures are considered their results may be considered more important than some other popular computer vision applications. 

This study provides an overview of computer vision studies of malaria diagnosis and intends to fill a gap in this area by doing so. There are some different interpretations of the requirements and thus the applicability of the proposed solutions to the problem. Here, these differences are addressed; the practicality, robustness, accuracy of the proposed solutions and their applicability to perform the actual diagnosis task are questioned. Moreover, the evaluation methods chosen to measure and assess the accuracy are discussed. In addition, some other works of the literature which concern the sub-problems or necessary sub-components are examined and placed in a general pattern recognition framework for the diagnosis application. The aim of this paper is to: 1) survey state-of-the-art of the methods concerning the problem; 2) describe a general computer vision framework to perform the diagnosis task; 3) resolve some ambiguities of different perspectives regarding the problem, and 4) point-out some future works for potential research studies.

Microscopy diagnosis is performed by manual visual examination of blood smears. The whole process requires an ability to differentiate between non-parasitic stained components/bodies (e.g. red blood cells, white blood cells, platelets, and artefacts) and the malarial parasites using visual information. If the blood sample is diagnosed as positive (i.e. parasites present) an additional capability of differentiating species and life-stages (i.e. identification) is required to specify the infection.

From the computer vision point of view, diagnosis of malaria is a multi-part problem. A complete system must be equipped with functions to perform: image acquisition, pre-processing, segmentation (candidate object localization), and classification tasks. Hence, the complete diagnosis system also requires some functions such as microscope slide positioning, an automated, fast, and reliable focus, and image acquisition. Some studies concerning image acquisition are examined in section **Image acquisition**. Usually, the acquired images from a microscope have several variations which may affect the process. These are usually addressed by pre-processing functions which are discussed in section **Image variations**. An important step in automated analysis is to obtain/locate possibly infected cells (i.e. candidates) which are the stained objects in the images. Detection of staining and localization of these objects are discussed in sections **Segmentation** and **Stained pixels and objects**.

In order to perform diagnosis on peripheral blood samples, the system must be capable of differentiating between malarial parasites, artefacts, and healthy blood components. The majority of existing malaria-related image analysis studies (e.g. [8-11,14,15,17]) do not address this requirement. This results in the over-simplified solutions, which are not applicable to diagnosis directly. On the other hand, the few methods which address the differentiation (e.g. [12,13,16]) have limited experimental results to show that their proposed solutions are comparable to manual microscopy diagnosis or able to replace it. To this effect the requirements for proper experimental data and set-up is discussed in section **Discussion**. In order to set the scene, a brief introduction about the malarial parasite, its species, and life-cycle stages is provided in the next section, followed by a short description of microscopy diagnosis.

### Malarial parasite

The genus *Plasmodium *has four species that can cause human infection: *falciparum*, *vivax*, *ovale*, and *malariae*. During the life cycle in peripheral blood, the different species may be observable in the four different life-cycle-stages which are generally morphologically distinguishable: ring, trophozoite, schizont, gametocyte. The species differ in the changes of the shape of the infected (occupied) cell, presence of some characteristic dots (Schüffner's dots, Maurer's clefts, Ziemann's Stippling) and the morphology of the parasite in some of the life-cycle-stages [3]. The life-cycle-stage of the parasite is defined by its morphology, size (i.e. maturity), and the presence or absence of malarial pigment (i.e. Haemozoin). Illustrations can be found in various sources, e.g. [3,18].

### Microscopy diagnosis

The WHO practical microscopy guide for malaria provides detailed procedures for laboratory practitioners [3]. Diagnosis initially requires determining the presence (or absence) of malarial parasites in the examined specimen. Then, if parasites are present two more tasks must be performed: 1) identification of the species and life-cycle stages causing the infection and 2) calculation of the degree of infection, by counting the ratio of parasites vs. healthy components (i.e. parasitaemia). However, these tasks are not necessarily performed separately or hierarchically.

**Figure 1:**
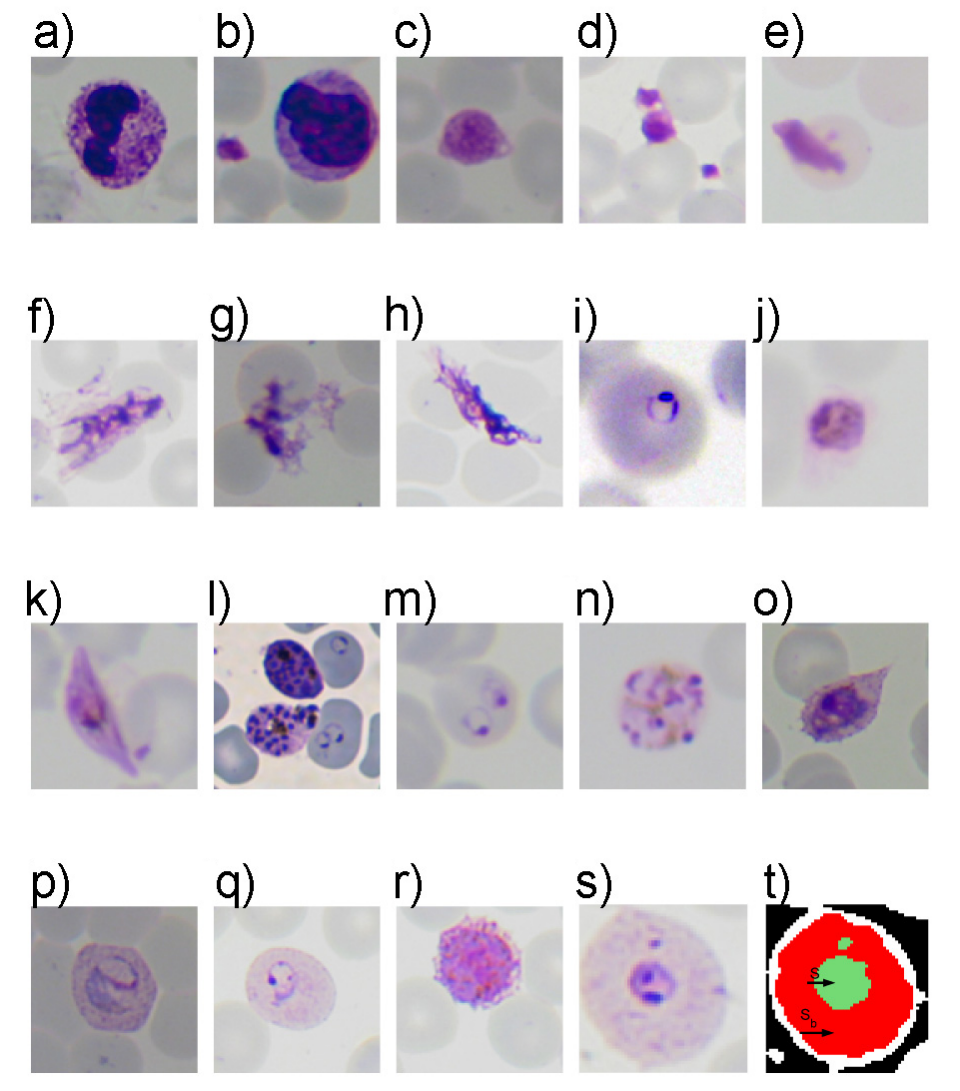
**Examples of stained objects**. (a, b) white blood cells, (c, d) platelets, (e)-(h) artefacts, (i)-(l) *P. falciparum *ring, trophozoite, gametocyte, schizont, (m, n) *P. malariae *ring and schizont (o, p) *P. ovale *and *P. vivax *trophozoites, (q, r) *P. vivax *ring and gametocyte, (s) *P. vivax *ring, (t) extracted stained pixel group. (*S*, green region(s)) and the stained object (*S*_*b*_, red region including the green one).

**Figure 2:**
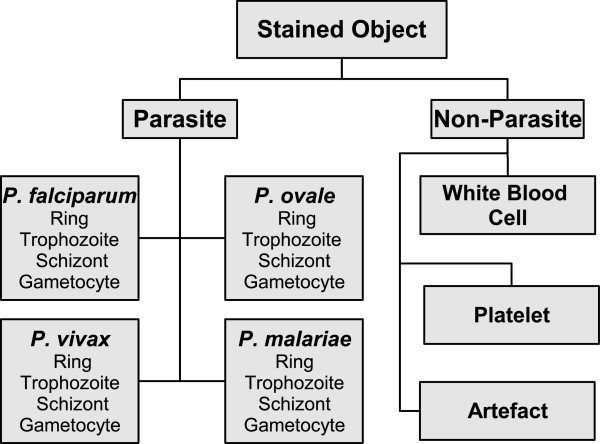
**Stained object classes: in a Giemsa-stained blood film an observed stained object can be a parasite from one of the four species of *Plasmodium *or a regular blood component such as white blood cell, platelet**. Artefact class represents bacteria, spores, crystallized stain chemicals, particles due to dirt, RBC anomalies (e.g. Howell-Jolly bodies, iron deficiency, reticulocytes), and other peripheral blood parasites.

Using a microscope, visual detection and identification of the *Plasmodium *is possible and efficient via a chemical process called staining. A popular stain, Giemsa, slightly colors red blood cells (RBCs) but highlights the parasites, white blood cells (WBC), platelets, and various artefacts (Figure 1). In order to detect the infection it could be sufficient to divide stained objects into two groups such as parasite/non-parasite and differentiate between them. However to specify the infection and to perform a detailed quantification, all four species of *Plasmodium *at four life-cycle-stages must be differentiated (Figure 2). Despite that the term 'artefact" is not very definitive, any stained object that is not a regular blood component or a parasite is referred here using this term: these include bacteria, spores, crystallized stain chemicals, and particles due to dirt [3]. It must be noted that other peripheral blood parasites and RBC anomalies (e.g. Howell-Jolly bodies, iron deficiency, reticulocytes) are included in this artefact class definition. They could be examined in individual dedicated classes if their identification is also required. 

A specimen for manual microscopy diagnosis can be prepared (on a glass slide) in two different forms: 1) a *thick blood film *enables examination of a larger volume of blood, hence it is more sensitive to detect parasites (as low as 50 parasites/*μ*l [19]). However, the thick film preparation process destroys RBCs and thus makes identification of species difficult. 2) On the other hand, a *thin blood film *preserves RBC shapes and parasites and is thus more suitable for species identification. A common practice in manual diagnosis is to perform positive/negative type decisions in thick blood films and identify species and life-stages in the thin films. Parasitaemia can be calculated in both types of smears [3].

**Figure 3:**
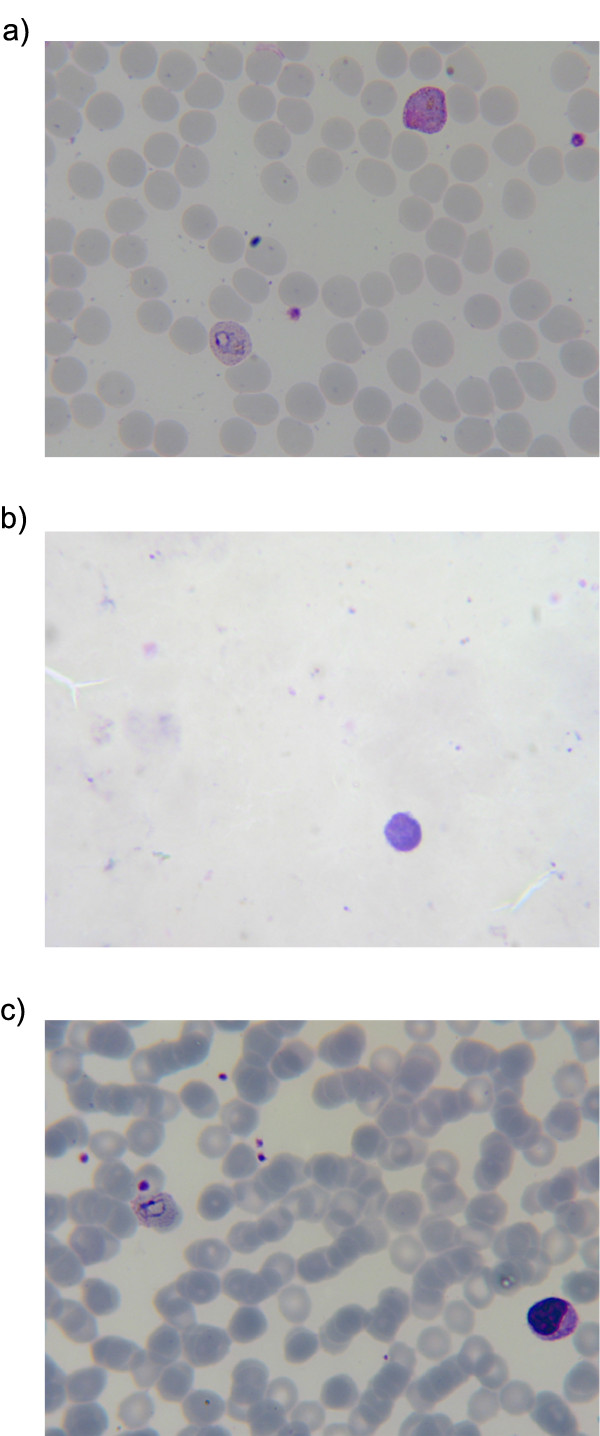
**Examples of Giemsa-stained (a) thin and (b) thick blood film smear images, (c) a concentrated (thick) field of a thin blood film smear**.

Figure 3 shows examples of stained thin and thick blood film images which contain malarial parasites. As far as this survey is concerned, almost all of the computer vision methods and related studies in the literature use thin blood film smears. Therefore, the discussions presented in this paper are on the thin film analysis works. However, the different requirements of thick blood films are remarked when appropriate. Polymerase chain reaction (PCR) methods are known to be more sensitive and more specific than (manual) microscopy [19-21]. Recent advances in the technique allow high-throughput applications and promote its use in routine diagnosis [22,23]. Mueller *et al *[24] show that Post-PCR ligase detection reaction fluorescent microsphere assay is more accurate than light microscopy in resolving species in the presence of mixed infections, which are common in the areas where malaria is endemic. PCR-based methods may replace microscopy examination as the gold-standard [20]; however, costs are significantly higher and more expensive instruments [25] are required.

On the other hand, emerging new technologies such as Rapid Diagnostic Tests do not require any special equipment and training. The detection sensitivity is lower but comparable to manual microscopy. However, they provide poor species discrimination and do not provide quantification of the results [26].

## Methods

There are many different paradigms of computer vision, which can be utilized to build an automated visual analysis/recognition system. Existing works on malaria commonly use mathematical morphology for image processing since it suits well to the analysis of blob-like objects such as blood cells. On the other hand, to differentiate between observed patterns statistical learning based approaches are very popular. The reader may find in this paper many technical terms that are used to explain different problems or approaches. Additional file 1 provides a brief definition for some of the image processing related terms (e.g. pixel, histogram, gradient), mathematical morphological operators (e.g. erosion, dilation, opening, granulometry), pattern classification concepts (e.g. feature, classifier, and training). More detailed information can be found in following sources: on mathematical morphology [27,28], on statistical pattern recognition [29-32], and on general image processing [33].

### Image acquisition

In [34] the required number of images to capture a 2 *cm*^2 ^region of specimen at 20× magnification is calculated to be nearly 1,300 images using a 1,300 × 1,030 pixel 2/3 inch charge coupled device (CCD sensor) camera. Diagnosis of malaria requires 100× objective magnification (recommended for manual examination), so the number of captured images would be 25 times higher. Hence, it roughly corresponds to over 30,000 slide movements, focus, and CCD sensor shutter operations which require a very fast technique. In order to reduce the time requirements, Wetzel *et al *[34] propose to capture the images while the slide is continuously moving, which introduced the problem of image blurring. They propose to use Xenon strobe lights instead of conventional lights to solve this problem, which probably raises the cost substantially.

It must be noted that a human expert will require more time to go through a slide and focus the microscope to observe 30,000 fields. Hence, the number of fields the expert would examine is usually smaller. In the WHO malaria microscopy tutorial [3], examination of only 100 fields is recommended before giving a negative decision. Additionally, in thick films, if a parasite is observed in a field, 100 more fields (or 200 WBCs, 0.025 *μ*l of blood) would be sufficient to calculate the parasitaemia. Since it is less sensitive, routine examination of thin blood films is not recommended for the positive/negative type of diagnosis. However, if parasites are found, examination of 50 fields (average 200 per field yields 10,000 RBCs in total) would be sufficient to calculate the parasitaemia in thin films. Thus, the speed requirements of the image acquisition system can be relatively easy to achieve. In addition, recently emerging fast focusing solutions and dedicated commercial slide scanning machines (e.g. US Patent No. 563437 filed on 2000-05-03) are promising to solve this important practical obstacle.

### Image variations

An image acquired from a stained blood sample (thick or thin) using a conventional light microscope can have several conditions which may affect the observed colors of the cells, plasma (background), and stained objects. These conditions may be due to the microscope components such as: the different color characteristics of the light source, intensity adjustments, or color filters. They may be due to the use of different cameras or different settings in the same camera: exposure, aperture diagram, or white balance settings. The differences in specimen preparation can cause variations as often as the imaging conditions [35]. For example, acidity (pH) of the stain solution can seriously affect the appearance of the parasites [3]. Addressing these variations can simplify the main analysis and contribute to the robustness of the system. In addition to the necessity of reducing these variations for the local process, if exchange of images and training samples could be made possible, then the different diagnosis laboratories which may employ the system in the future may benefit from a uniform diagnosis expertise.

### Illumination and thresholding

Most microscopes are equipped with (calibration) components to provide uniform or relatively uniform illumination. A common illumination calibration standard is *Kohler Illumination *named after its inventor August Kohler [36]. In this method, transmitted illumination from the light source is aligned and focused for a parallel and uniform illumination. This is often neglected by microscopists since the human vision system is adaptive to local illumination changes, however for an image analysis algorithm variations can cause serious problems.

Uneven illumination can be simply dealt with by acquiring a separate image of illumination to subtract from images later (e.g. an empty field on the blood slide [37]). However, for a particular test image coming from an external source, the imaging system may not be accessible to record a reference image of illumination. An alternative method is to filter the images to remove the variation in the illumination. In the case of a smooth varying illumination, as in most microscope images, a filtering operation may reduce the potential effects. This may be performed by applying a Gaussian filter [38] or morphological image filtering method [28]. For example, for the blood slide images the smooth varying content can be calculated with morphological *closing *(on the grey scale image) by a sufficiently large (than the target objects) structuring element [37]. The "sufficiently" large structuring element size can be determined using *area granulometry *(described later in **Scale and granulometry** section, see also [39]).

Halim *et al *[14] proposed to correct uneven illumination by calculating gradients in the polar coordinates (*r*, *θ *coordinate system) of the background image which was calculated by simple thresholding. However, in some cases the illumination can be excessively uneven and hinder a thresholding operation. Ross *et al *[16] employed Otsu's thresholding method [40] to obtain a binary foreground-background representation; however, this method also performs global thresholding and is probably negatively affected by uneven illumination.

Rao [8] proposed the use of mathematical morphology to produce foreground binary masks in the presence of uneven illumination. The proposed method performs an initial rough thresholding to separate foreground and background histograms from which two separate threshold values are found. In the final step, the morphological *double threshold *operation [28] is employed to obtain a refined binary foreground mask. However, it was shown in [37] that due to the final global threshold operation even this method is not immune to uneven illumination, and that the illumination must be corrected prior to any global (thresholding) operation.

### Color

The different *Plasmodium *species are distinguishable from each other and regular blood components and artefacts by their characteristic shapes (morphology) and color properties [3]. If the color-based properties of the images are used then color variations must be addressed.

Various studies address the calibration and color constancy issue in imaging for general machine vision purposes [41]. It is however, difficult to say the same for microscope imaging which requires special approaches. The difference with microscope imaging is that calculations based on the Lambertian surface model and use of the reference color charts are not appropriate because the sensor (or human eye) does not receive the light reflecting from a surface. The light reaching the sensor is the attenuated light which is left after the object's (i.e. specimen's) absorption. In fact, image formation of the stained slides with light microscopes are more appropriately modelled with the "Beer-Lambert Law" which states that there is a linear relationship between the concentration, thickness of illuminated media, and the "absorbance" [42]. Additionally, the reference color patches (as proposed for other medical imaging applications, e.g. [43,44]), are not practical for microscopes. Even though it was possible to manufacture them; there is still the human factor in preparation of the blood film slides which results in non-standard and non-homogeneous staining concentrations and appearances [45].

The problem of non-standard preparation of the blood film slides (specimen) was addressed in [46]. To correct under/over staining conditions of the slide, they obtained the spectral transmittance by a multispectral camera (a camera equipped with different filters to capture the spectral reflectance on separate bands). They mathematically modelled the relation between the transmittance and the amount of stain (dye) for each pixel using the Beer-Lambert Law and Wiener inverse estimation [42]. This [46] is an important study providing a mathematical model of the staining concentration-transmittance relation, which enables digital correction of non-ideal stain concentrations. However, the variations due to the different camera parameters and light sources were not addressed which leaves the imaging side of the problem fuzzy. Nevertheless, the malaria diagnosis system may not have the luxury of adding the cost of a multispectral camera; it is not practical to capture many (e.g. [10]) different bands of the same field to estimate the amount of dye.

In [37,47] the authors proposed a practical method which exploits the special characteristics of the peripheral thin blood film images that are easily separable into the foreground and background regions. After separation, the method employs the simple grey world assumption in two consecutive steps to provide an effective color correction. However, the method is not directly applicable to thick film analysis due to the assumption of an expected foreground scene.

### Scale and granulometry

In healthy human peripheral blood, the average diameter of an RBC and platelet is between 6–8 *μ*m and 2–3 *μ*m, respectively. WBC size can vary between 8–20 *μ*m depending on the type [48]. The CCD pixel resolution and magnification (i.e. field of view) can be used to calculate expected sizes of the blood cells that are present in the image. Moreover, this information can be used to calculate the image pixel scale in physical units. However, the magnification information may not be accessible or the imaging set-up may not be present. Additionally, there are some conditions (e.g. anaemia) which result in abnormal cell shapes and sizes [48].

Almost none of the methods which aim at diagnosis of malaria or related processing tasks are concerned about the actual physical scale of the objects in the processed images, but the size of the cells in the image plane to enable scale-independent processing since the cell size information used as a parameter in many algorithms.

The granulometry of mathematical morphology [49] (pattern spectrum) can provide the size distribution of an input image. It is computed via a family of *openings *which have increasing, anti-extensive, idempotence properties [50]. Though the definition of granulometry does not suggest any special type of opening operation, in practice it is usually implemented via a set of increasing-width structuring elements of a fixed pattern (e.g. square, disk, and hexagon). Granulometry of a grey level image *X *can be calculated as follows:

(1)

where *γ*_*Si*_(*X*) is morphological opening of image *X *using structuring element *S*_*i *_∈ {*S*_1_,..., *S*_*N*_}, and *m*_0_ sum of pixel grey level values in the input image.

The differential form is usually called a *pattern spectrum*:

(2)

A granulometric analysis to estimate the size of the cells of the input image (i.e. RBCs) was proposed in [51,52] using circular structuring elements. Later, they used granulometry-based size estimation in consequent studies for segmentation [53] and analysis of malaria infected blood cell images [10]. Similarly, [16,54] employed granulometry using fixed shape structuring elements for RBC size estimation. However, the abnormal conditions which cause irregular cell shapes and the holes inside the cells degrade the accuracy of this method.

Rao *et al *[55] proposed to employ *area closing *for closing the holes inside the cell regions to improve the granulometry performance. Later, to cover deformed non-circular shapes of cells, they [39] proposed to employ the *area pattern spectrum *which was then used in different studies as an improved size estimator for the blood cell images [8,56,47]. In these studies, the area pattern spectrum was calculated with a series of *area openings *as in (3), which is highly inefficient and time consuming.

(3)

where  is morphological area opening of image X with area threshold *λ*_*i *_∈ Λ = {*λ*_1_, *λ*_2_,..., *λ*_*n*_}. Area opening is attribute based and hence a structuring element is not used; however, connectivity and area threshold *λ*_*i *_must be defined (see additional file 1 and [57] for definitions). Note that the choice of different connectivity can affect the noise sensitivity of the morphological operations and may affect the results [58]. A single pass algorithm of area granulometry was originally described in [59]. A method for fast computation (independent of the number of scales) was proposed later in [60]. This method is based on a connected sets tree (i.e. Max-Tree [61]) representation which allows computation of non-increasing attributes as well [62]. A recent study on the attribute granulometries can be found in [63].

**Figure 4:**
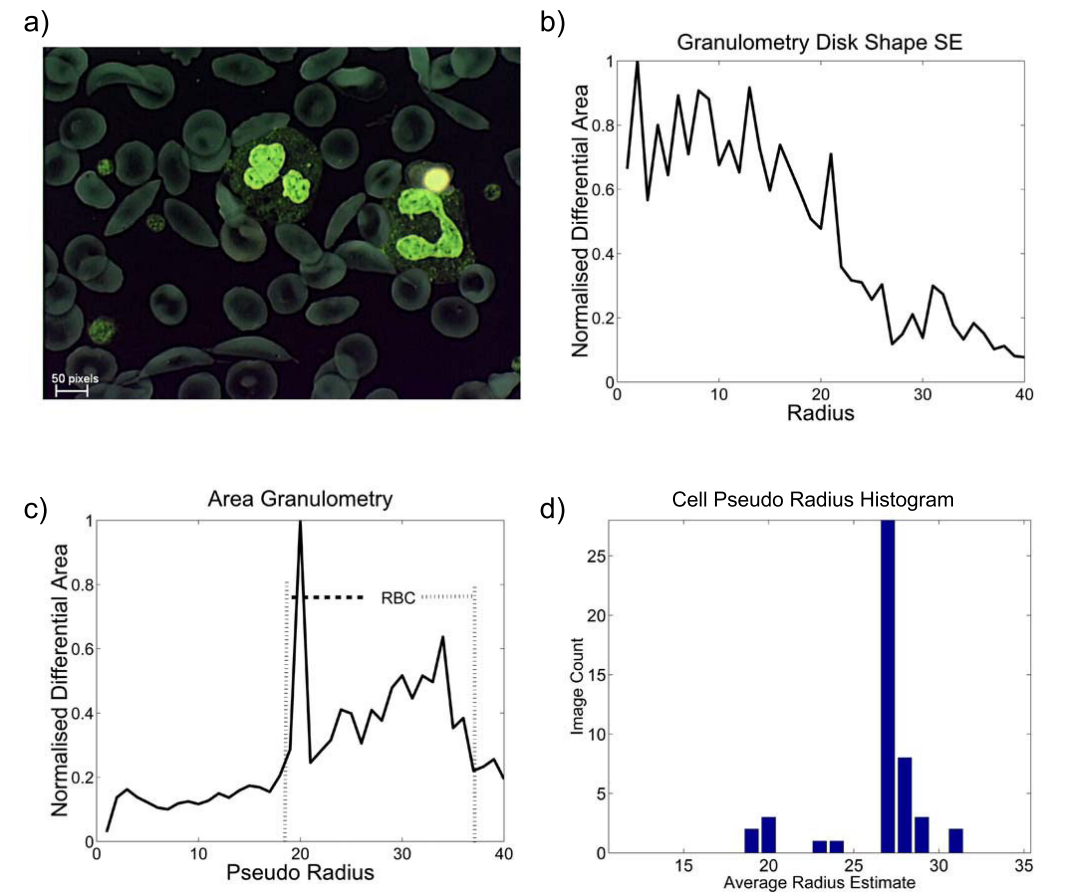
**Size granulometry vs. area granulometry**. (a) negative image of the grey level sickle cell image, (b) granulometry using disk shaped structuring elements, (c) area granulometry, (d) area granulometry based cell size estimation varies in different fields of a thin film although the magnification is constant.

To provide a comparison, Figure 4 shows the plots of granulometry and area granulometry calculations on the grey level negative of a thin film image of a specimen with sickle cell condition (irregular cell shapes). In this image, the RBC diameters changes between 20–35 pixels. Since RBCs are not all circular and homogeneous (they may have holes), size-granulometry, which uses circular structuring elements, could not produce an informative result. On the other hand, area granulometry is more accurate because there is no assumption on the shape of the cells and it has the computational advantage because it can be computed within a single pass and independent of the number of scales [63]. Therefore, it should be preferred to granulometry with the fixed shape structuring elements.

### Average cell size estimation

A common practice is to estimate average cell size with the peak index of the granulometry (which can be an area or radius index). This assumes that the thin blood film image is covered by resolvable individual RBCs of similar size. However, the RBC size variation in normal blood and the disorders which cause abnormal RBC sizes are neglected. In addition, the thickness of the thin film varies through a slide and this results in varying focus depths, which can also change the calculated average cell area. Figure 4(d) shows the average cell pseudo radius (estimated by the peak index of area granulometry) distributions that are calculated from images of 140 different fields of a single thin blood film specimen. The optical magnification is the same for all the images, however estimated average cell size varies remarkably. This is simply caused by the overlapping cells in the dense image fields and the differences of the field thickness. Hence, the size estimation that is based only on the area granulometry peak is not very precise for thin blood images. Existing malaria diagnosis methods concentrate only on using size or area granulometries. However, the granulometry concept has more potential to explore, which may be applicable to blood film image analysis. In [59], Breen and Jones extended the definition of granulometry to be calculated with any set of attribute openings or non-increasing opening-like operations: thinnings [28]. In [62] Urbach *et al *proposed an implementation of *shape pattern spectrum *which was later extended to the calculation of 2D granulometries (Shape × Area) in [64] and to the vector granulometries in [65].

A final remark is that it is possible to calculate size distribution in thick blood film images (Figure 3(b)) using area granulometry. However, it is difficult to use an "average object concept" for these images because RBCs are destroyed and not observable. Furthermore, it is not guaranteed that the observed field will contain any well-defined structure, e.g. WBCs, platelets.

### Segmentation

Probably one of the most common shared tasks in image analysis systems is segmentation. Segmentation aims to partition the image plane into meaningful regions. The definition of the meaningful regions and partitioning method is usually application specific. For example, the methods can be aimed at separating foreground-background, moving-still regions or objects with specific properties from the scene. The segmentation strategy can be a hierarchical partitioning that operates deductively to define first a higher level of object plane, then the objects, and then sub-object components. The inductive approaches define first the objects of interest with a specific property then perform higher levels of partitioning(s) if necessary. In order to localize highlighted (stained) objects, either inductive or deductive segmentation approaches can be followed. In some studies [10,16,37] first the stained objects were identified by their intensity and color properties; then only the RBC regions containing the stained objects were segmented from the image. On the other hand, in some studies, e.g. [8] a deductive strategy was followed: the image was first separated into foreground and background regions; then foreground regions were segmented to obtain individual RBC regions; then these were further analyzed to detect the presence of staining. The global segmentation procedure is applied usually if a deductive approach is proposed.

The common problem associated with the segmentation of thin blood film images is the under/over-segmentation of the cells. Under-segmentation, i.e. including two or more cells in one region, is usually caused by unresolvable cell boundaries of contacting or overlapped blood cells. On the other hand, over-segmentation, i.e. dividing a single cell to more than one region, can be related to heterogeneity of the cell region or incorrect assumptions of the cell size. Several techniques have been proposed to prevent under/over-segmentations in thin blood film images: morphological gradient [53]; morphological area closing and distance transform [66]; area top-hats [8]; Bayesian color segmentation and watershed segmentation [67]; minimum area watershed transform and the circle Radon transformation [56]; template (ellipse) matching [14]; multi-dimensional Otsu thresholding [68], and clump splitting [69].

Unfortunately, none of these methods are applicable to highly concentrated fields of thin blood film images (e.g. overlapping cells, see Figure 3(c)). Hence, either the whole analysis must be constrained to process only the "segmentable" (i.e. lightly concentrated) fields or global segmentation must be totally avoided. It is possible to evaluate the image's (field's) cell concentration or density using the granulometry-based method in [54]; however eliminating some fields from the analysis may degrade the sensitivity of the overall system.

A global segmentation approach can be replaced with localized analysis which is discussed in the following section. However, another purpose of the segmentation is to count individual RBCs, especially for the quantification of infection, i.e. parasitaemia calculation. It may be possible to estimate the RBC count without performing a perfect segmentation of the image [54]. Alternatively, parasitaemia can be calculated with respect to WBC count rather than RBC's [3] if they can be identified.

### Stained pixels and objects

The staining process highlights the parasites, platelets, WBCs, and artefacts in a thin blood (peripheral) film image. In order to analyze the highlighted bodies it is essential to identify the pixels and thence locate the object regions. However, it must be noted that other blood parasites [70] and some disorders of blood, e.g. iron deficiency are also highlighted by the Giemsa-stain.

Some methods of the literature name and describe this step as "Parasite Detection" (or parasite extraction). This results in over-simplistic solutions which are not applicable to diagnosis of malaria, because diagnosis must be performed on actual peripheral blood specimens of the patients which are certain to contain other stained bodies: WBCs, platelets and artefacts and may be infected by other parasites or may have other disorders (e.g. iron deficiency). This may be related to the use of *in vitro *samples as for the experimental data. Usually *in vitro *culture images consist of samples grown in a laboratory environment. Hence, they are cleaner of artefacts and do not contain platelets or WBCs. In [13,16,37] the necessity of differentiating parasites and other stained bodies was addressed. Therefore, since it defines the process more clearly, the term "stained objects" is used; and different methods to find and extract them are discussed here (instead of "parasite detection"). A simple example of the stained pixels and stained object relationship is shown in Figure 1(s–t).

Di Ruberto *et al *[10] employed morphological regional extrema [28] to detect (i.e. marked) the stained pixels, then used morphological opening to extract the object regions marked by these pixels. However, they identified the WBCs, platelets, and schizonts by comparing their size to the average cell size obtained from granulometry and exclude these from further processing. Hence, their method can be regarded as addressing the detection issue. However, detection of stained pixels with regional extrema is error prone because it will locate some pixels even if the image does not contain any stained pixels. Moreover, eliminating WBCs and platelets with respect to the average area value can eliminate some parasite species which enlarge the RBCs that they occupy. For example, *Plasmodium vivax *infected cells can enlarge up to 2.5 times [3]. Ross *et al *[16] used a similar approach: they have used a two level thresholding (global and local) to locate stained pixels, then used morphological opening to recover the object binary masks. Both of the methods rely on opening and disk shaped structuring elements which creates problems because the cells are rarely perfect and flat circles.

Rao *et al *[8] used thresholding to detect stained pixels, however they pre-processed the images to remove a global bias color value that is caused by staining, which is to prevent false pixel detections if the image do not contain any stained pixels. Since they use global segmentation to locate individual RBCs, the stained objects are defined by the regions which contain stained pixels. As stated in the previous section global segmentation is error prone, unless examined fields are limited to the lightly concentrated fields. In addition, it must be noted that employing a thresholding operation to detect stained pixels assumes an ordered relation between stained and un-stained pixels, e.g. "stained pixels are darker than others". 

The authors [13] proposed to detect stained pixels according to their likelihood where a pixel's red-green-blue color triple was used as the features and stained and un-stained classes were modelled using 3-d histograms. This removes the limitation of the "stained pixels are darker/brighter" definition. Using the detected stained pixels as markers, they located the objects by using morphological area top-hats and reconstruction [28]. This approach prevented over-segmenting of stained bodies, which could be caused by employing global segmentation based on area heuristics.

Detection of stained pixels is not a very complex problem especially with the use of color correction algorithms. However, as pointed out in [37], one of the biggest problems of thin blood film analysis is to locate the stained objects and define their boundaries, because the stained pixels which are used as markers may be due to a variety of objects, e.g. to an artefact which can be any size or shape [3]. In addition, even for the defined blood components, the process is error prone because the boundaries are not always resolvable, especially in highly concentrated image fields (Figure 3(c)).

One alternative, which may worth an investigation, is to locate the stained pixels by some method and, avoiding object localization, to use directly a sliding-window approach on these regions to produce queries to a classifier. The sliding-window approach, usually in multi-scale, is used successfully in many general pattern recognition applications, e.g. face detection [71]. The size of sliding-window can be determined with respect to the physical scale information; alternatively area granulometry based average cell size estimation can be utilized. In addition, the sliding-window approach may be a generalized solution to both thin and thick film analysis problems. Thick blood films do not have resolvable RBCs. Hence, the detected stained pixels are isolated; and do not allow further defining operations based on the shape and size assumptions. Moreover, it may be more practical to adapt this method for detection of other blood borne parasites and disorders, which may be in any size or shape.

### Classification

There are only few studies which propose a classification procedure [13,16,37] to differentiate between parasites and other stained components or artefacts. The method described in [14] also proposes a classification to differentiate between a healthy RBC and an "infected" RBC. However, from the diagnosis point of view the essential task is to identify parasites in the presence of other stained structures, artefacts, and then finally identify the species. As in Di Ruberto's [10], the approach to the classification task in a recent work also was also limited to detection white blood cells and gametocytes by area information, for the purpose of excluding these from parasitaemia calculation [72].

However, although they do not address the parasite/non-parasite differentiation, some automated diagnosis of malaria studies rather focused on the life-cycle stage classification. Di Ruberto *et al *[10] proposed to use the criteria of circularity (measured by the number of morphological skeleton endpoints [28]) and color histogram to classify the life-stages into two categories: immature and mature trophozoites. Their test set contained 12 images. Rao *et al *[8] proposed a rule-based scheme (area and haemozoin existence) to differentiate five life-stages. They experimented on a set of *Plasmodium falciparum in vitro *samples which contain immature-mature trophozoite, early-mature schizont but no gametocyte class or other types of stained object.

Ross *et al *[16] proposed a consecutive (detection-species recognition) two-stages classification for the problem. They proposed to use two different sets of features for parasite detection and species recognition. The initial feature sets were comprised of many color- and geometry-based features. For example, they have used average intensity, peak intensity, skewness, kurtosis and similar abstract calculations from the red green blue channels together with the same calculations from the hue-saturation-intensity channel images. For geometrical features, they have identified roundness ratio, bending energy, and size information, i.e. area, in their feature set. For parasite detection and following species recognition tasks, the initial feature sets were comprised of 75 and 117 features, respectively. Using principal component analysis [32] they reduced the number of features to 37 and 38, for detection and species recognition respectively. They have trained a two level Back Propagation Neural Network for parasite detection and species recognition. The results for detection were reported as: sensitivity (SE) of 85.1% with a positive prediction value (PPV) of 80.8%. The specificity value or false detection rate was not reported. See additional file 2 for descriptions of these measures. For the species recognition task the SE-PPV results were: *P. falciparum *57%–81%, *P. vivax *64%–54%, *P. ovale *85%–56%, *P. malariae *29%–28%. The life-stage recognition problem was not investigated. Their experiments used a training set comprised of 350 images containing 950 objects and in the similar test set.

The authors proposed a KNN based parasite detection scheme in [13]. Later, the study was extended for a combined analysis of detection, species, and life-stage recognition [37]. They studied more generic features such as indexed color image histogram, correlogram [73], Hu moments [74], and localized area granulometry (3), and proposed a concatenated feature vector. The results for detection were SE:72.1%, PPV:85.1%, specificity SP:97.45%, and negative prediction value NPV:94.52%. However, to reduce the effects of class imbalance on the results they proposed to use a biased KNN classifier [75]. Using receiver operating characteristics (ROC) analysis [76] they showed that an adjustable sensitivity-specificity detection performance can be provided. The adjustable scheme is valuable because the methods [37] (also as in [16]) report their results based on per-object accuracies rather than the per-specimen accuracy which would be expected by a medical diagnostic test. This difference is discussed in more detail in **Discussion **section. For an expert microscopist, the tasks of parasite detection, life-stage, and species recognition are not necessarily hierarchical, sequential, or independent. The diagnosis expert can perform all these tasks in a single classification or sequentially or even partially depending on the discriminative information that exists in the observed object. For example, the expert can recognize a *P. falciparum *ring-stage parasite directly; or recognize a ring stage parasite and then can seek for more discriminative parasites to decide its species category. Moreover, it may not be required to determine the life-stage of every single parasite because a thorough examination of the whole slide can reveal the most frequent life-stage and the condition of a single/mixed species infection. However, in manual practice, parasitaemia calculation requires counting of parasites that are not gametocytes. Therefore, if one considers the diagnosis of a whole slide, the detection, species, and life-stage recognition tasks can be regarded as contextual. It may be possible to incorporate the contextual information into the classification for malaria diagnosis, as proposed in [77] for WBC disorder detection.

For species recognition and life-stage recognition, the authors [37] followed a different approach and compared joint and separate classification schemes, which concluded that parasite detection can also be performed with a joint classification (20 class all or 16 class parasite only classes) instead of a separate two-step classification scheme (e.g. binary detection followed by four class species recognition). In the 20-and 16-class classification schemes, species and life-stage recognition results were comparable to manual microscopy [4,78]. However, it must be noted again that these results were based on a single observed object not a whole specimen which may have thousands of objects. 630 images containing 4,000 objects were used in *hold-out *evaluation for detection and *leave-one-out *evaluation for species and life-stage recognition experiments.

Nevertheless, the joint classification scheme, removing the necessity for a binary detection (parasite/non-parasites classification), may improve the expandability and scalability of a diagnosis system by preventing a narrow reference to "parasite" and "non-parasite" classes. For example, if restricted to perform a binary detection, a malaria diagnosis system will have a different notion of "parasites" than a diagnosis system for Babesiosis or Trypanosomiasis which are examples of other peripheral blood parasites [70]. However, a multi-class joint classification scheme will treat each species and life-stages as separate and provide other parasites or conditions to be handled by the system. This should be supported by the use of generalized features instead of the optimized features.

## Discussion

### Imaging

In order to be feasible for mass screening or diagnosis, a computerized inspection system must be provided with the automatic slide positioning and image capture facilities. In performing diagnosis of a single sample, the slide must be re-positioned at least 100 times, focused, and captured. The system would be highly impractical if manual assistance was required. Some of the state-of-art microscopes that are located in well-equipped laboratories already can provide these functionalities. However, one of the general aims of malaria diagnosis research should be to produce a cost-effective diagnosis method which can be used especially in the economically weaker areas where malaria is endemic and causing a serious number of deaths. A possible solution to this problem can be the dedicated slide scanning boxes which have already some examples in the market. A customized slide scanning system does not require many of the general-purpose functions of a microscope, but a highly sophisticated automated focus technology and hence at the moment existing products are far from meeting the criteria of being low cost. However, for some of other applications which can be performed semi-automatic such as training, research, or tele-diagnosis these requirements may be easier to meet. Simply any optical compound microscope with a digital consumer camera can be used as an imaging system to acquire blood smear images [37].

### Stained object detection and localization

Existing methods based on segmentation are not applicable to all fields of a blood slide (negatively affected by relative thickness). Hence, either only the lightly concentrated fields should be processed or this strategy should be altered. The alternative stained object extraction methods based on local morphological processing are mainly heuristic and are partial solutions. In addition, they are highly specific to malarial parasites. A possible unified solution could be an adaptation of the sliding-window-based approach which has been successfully used in other general object detection problems [71,79]. This can be a generalized solution which is applicable to thin and thick blood films and for detection of other blood parasites. In this case, a multi-scale scale search can be performed [79,80]. Otherwise a sophisticated problem that could arise is how to determine the search window size. Area granulometry can be used for this purpose in thin blood films; however, as shown, it may not be very precise to detect scale. Nevertheless, some works which concern granulometries of other (and joint) attributes [63,64] can guide future research efforts to improve scale determination in thin blood films.

### Sample independence

The choice of the testing procedure and error measures can significantly affect the results [81]. In practical pattern recognition, a general practice is to estimate the accuracy of the overall procedure by performing tests on a concrete set of samples. The factors affecting the results are complex and unfortunately the true error rate is unknown. Among several commonly used testing procedures in pattern recognition the *hold-out *or *leave-one-out *evaluations ensure the independence of the training and testing samples, and thus may suggest a generalization in real applications [32]. In the hold-out evaluation, data is randomly separated into two sets, training and optimizations are performed on one and the generalization performance is tested on the second test set. In the leave-one-out evaluation, in order to test each sample of a set of *N *samples, the remaining *N *- 1 samples are used for training. The procedure is applied *N *times for each sample in the set. Leave-one-out is the marginal case of the *m-fold *evaluation where *m *= *N *- 1. A detailed discussion on the hold-out and leave-one-out evaluation methods can be found in [81].

However, the sample independence should be more carefully examined in order to discuss the capabilities of a potential system. Specific to diagnosis, it is possible to define different levels of sample independence [37]. Ideally, a system should be tested with different samples which are obtained from (1) different images, (2) different specimens (blood films), and (3) different imaging sources (e.g. laboratories, hospitals) to simulate the diagnostic generalization capability. In addition, the test set should be allowed to contain completely healthy specimens (negatives) and specimens of other conditions, e.g. iron deficiency, WBC disorders, or other parasites. Having a sufficient degree of independence between training and test samples, one should be careful to avoid repetitive tuning and optimizations in a fixed sample set [82].

### Per-object vs. per-specimen results

The average sensitivity threshold for manual microscopy (by an expert microscopist, using a good microscope in good working order) of thick blood film examination is reported as 50 parasites/*μl *of blood [19] whereas thin blood films are reported to be 1/11 less sensitive [83]. Let us assume the thin blood film sensitivity of *expert *microscopy as 500 parasites/*μ*l. This corresponds to 0.01%, based on the fact that an average blood sample contains 5 million RBCs per 1 *μl*. The expert sensitivity threshold (500 parasites/*μl*) is based on the assumption that an expert microscopist works with 100% per-object sensitivity (i.e. the expert is assumed to always recognize the parasite correctly), however, he/she can examine only a limited number of fields in a limited time. For example, if the microscopist examines 100 fields with an average of 200 RBCs, he/she would be able to see only 20,000 RBCs which would have 86.52% probability of seeing an infected RBC in a specimen of 500 parasites/*μl*. In order to ensure that a higher probability (e.g. *P > *0.999) of observing an infected RBC, the expert should observe at least with 45,837 RBCs (i.e. 229 fields). From the same perspective, if an automated parasite detector's (e.g. [37]) per-object sensitivity is ~72.37%, it will require at least six parasites to be observed to ensure that at least one parasite is detected, which can be found in 45,889 (*P > *0.999) RBCs if the specimen has 500 parasites/*μl*. On the other hand, the specificity value (~97.45%, [37]) of the same detector would produce ~114 false positives (detections) where 45,889 RBCs are observed. Therefore, the per-object performances may be required to be higher to be comparable to the manual microscopy. Another interpretation is that the classifier of sensitivity 72.32% and specificity:~97.45% would be limited to operate only on the higher parasitaemia levels.

It should be noted that the routine diagnosis is not performed on thin blood films. A study of routine malaria diagnosis in the UK showed that the average detection sensitivity for microscopy was around 500 parasites/*μl *[78]. This would correspond to 5,000 parasites/*μ*l in thin blood films if the 1/11 sensitivity ratio as given in [83] is considered. It should be also considered that although it was assumed that the *expert *microscopist works with 100% per-object sensitivity, a recent study shows that the agreement rate among even the reputed expert microscopists is not 100% and is negatively affected by the lower parasitaemia levels [84].

Nevertheless, a large-scale test which contains many specimens (positive-negative, mixed, other parasites, other blood disorders) can provide a useful evaluation of the diagnostic tests. In practice, the requirements of the evaluation and sample independence to prove a medical diagnostic test's clinical practical value are much higher [85].

### Thick film analysis

As far as this survey was performed, only a preliminary study [86] for thick blood film analysis was found in the literature. The thick film examination sensitivity in microscopy diagnosis of malaria is higher than for thin films: 50 parasites/*μ*l [19]. However, species recognition is more difficult due to destroyed RBCs and deformed parasites. Hence, for this task thin blood film examination is being used. If the process for detection in thin blood films could be made fast enough it can screen more fields to reach an increased detection sensitivity threshold to match that of thick films. For example, hypothetically, processing 500 fields (including average 200 RBCs) instead of 50 (recommended for manual microscopy) can reduce the detection sensitivity threshold by a factor of 10. This, which can be empirically tested by a large-scale test, can show that thick blood film analysis may not be essential and eventually remove the necessity to prepare and examine a different blood film. On the other hand, it would be a great improvement to microscopy diagnosis of malaria if the same processing speed can be achieved in thick film analysis and thus the sensitivity threshold can be reduced to a level below the expert microscopist's performance (i.e. ~50 parasites/*μ*l [19]).

## Conclusion

This paper provides a good basis for researchers who are starting to investigate the automated blood film analysis for diagnosis or screening of malaria or similar blood borne infectious diseases. In this paper, a review and critique of computer vision and image analysis studies which address the automated diagnosis of malaria on thin blood film smears and its necessary auxiliary functions is provided. The computerized diagnosis of malaria is addressed at system level; its practicality is discussed by pointing at the issues of imaging, its interoperability is emphasized by addressing variations which can be caused by different imaging set-ups or differences in specimen preparations. A system would benefit from the capability of processing images of external sources or allowing exchange of images and learned parasite models, at the same time its functions may be calibrated for the imaging equipment that it operates on. An open problem is the automated analysis of thick films, despite being more sensitive in detection, has not been investigated from a computer vision perspective. In the existing thin film analysis literature there are some works which propose oversimplified solutions that are not applicable to diagnosis. For the studies which are methodically applicable to diagnosis, some limitations arise from relying on global segmentation of the image. An alternative sliding-window-based detection approach [79] (avoiding segmentation) could be a generalized and possibly better solution to the problem and may be applicable to both thin and thick film analysis.

In addition, the difference between the per-object and per-specimen detection results is emphasized. The evaluations which are currently based on per-object are not necessarily meaningful from the clinical perspective. The existing and prospective methods must be evaluated on large-scale specimen sets and results should be reported based on per-specimen (e.g. per-film) sensitivity and false positive detection rates. Moreover, sample independence of the experimental data must be taken into account and the test data should include a variety of peripheral blood samples including both negative and positive specimens with different levels of parasitaemia and preferably should be acquired using different imaging sources. Finally, future work should also consider expandability and thus the applicability to other blood-borne parasites and disorders.

## Competing interests

The authors declare that they have no competing interests.

## Authors' contributions

FBT structured the review and wrote the paper. AGD and IK reviewed the submitted manuscript and contributed to the writing of the paper. All authors read and approved the final version.

## Supplementary Material

Additional file 1 **Description of image analysis and pattern recognition terms.** This file provides brief explanations of image processing and pattern recognition related terms. Click here for file

Additional file 2 **Description of the performance measures**. This file provides explanations of the accuracy related terms. Click here for file
