# Supplementary material for: Computer vision for microscopy diagnosis of malaria
Source: Malar J. 2009 Jul 13;8:153. doi: 10.1186/1475-2875-8-153 (PMC2719653; doi:10.1186/1475-2875-8-153)
Supplement: Additional file 1 — Description of image analysis and pattern recognition terms. This file provides brief explanations of image processing and pattern recognition related terms. [file 1475-2875-8-153-S1.pdf]

Additional file 1
Description of image processing and pattern recognition terms

General image processing

Some of the common terms which are used in the paper are described below. Extensive information on image processing and analysis is available in various texts, e.g. [1, 2]

- **Pixel**
  Computers operate on digitized images, which are mappings from continous three dimensional world to discrete grids. The elements (points) on the grid are called pixels, which represent mapped intensity values (i.e. irradience).

- **Neighbourhood**
  A digital image is formed by pixels that are located in a grid (usually rectangular). The set of adjacent locations of a pixel is called its neighbourhood, which can be redefined according to the particular processes, such as filtering.

- **Filtering**
  Filtering is a general term to describe the process of removing some defined property or quality from an image. A simple filtering operation can be performed by a convolution, which is to calculate the transformation that is described by a filter in every pixel location of the image. Different filters (kernels, masks) can be used to smooth the image (e.g. Gaussian smoothing), locate edges in it, change the colour representation etc.

- **Gradient**
  Gradient of an image is the rate of change of the intensity in each pixel location in a certain direction. The change is due to the pixel's intensity difference to its neighbour pixels. This can be calculated by several ways, e.g by subtracting adjacent pixel values along rows or columns.

- **Histogram**
  Histogram is the count of occurences of pixels in different levels. It provides useful information about an image by representing its pixel value distribution. Since different structures have different pixel values, it can be used to separate or identify main structures in the image such as foreground and background regions.

- **Thresholding**
  Thresholding is the operation of mapping the image pixel values to binary values (e.g. 0,1) according to their comparison with a specified value (threshold). A histogram can be used to determine the threshold values automatically. For example for the foreground/background separation, if the objects are brighter than the background, pixel values greater than the threshold value can be assigned to 1, otherwise 0, to produce a binary mask.

Mathematical morphology

Mathematical morphology (MM) is a theory concerned with morphology or spatial analysis of images. It is based on set theory and lattice algebra [3]. Many functions provided within MM are based on non-linear transformations in which the output is given as the relation between input set and a transforming set which is defined by a structuring element or an attribute. The attribute can be based on an increasing measure such as width of a basic shape (square, circle etc.), area or can be non-increasing measure such as circularity, elongation, etc. The theoretical details and more information can be found in texts [4, 5].

- **Connectivity**
  Connectivity in a digital image can be explained with graphs. A connected graph is the graph in which any two of its vertices can be linked by a path. For example, if two sample points $[(x1, y1), (x2, y2)]$ in a digital image are taken as vertices: these vertices are said to be 4-connected if $|x1-x2|+|y1-y2| = 1$, whereas they are said to be 8-connected if $max(|x1-x2|, |y1-y2|) = 1$. The connectivity definition is required to define the neighbourhood of a specific coordinate in the image plane. Almost all the MM operations can produce different results with different connectivity definitions, e.g. [6].

- **Structuring element**
  Structuring element can be defined as a set which modifies a morphological operation's behaviour with a defined attribute (e.g. shape, size). In its simplest form, a structuring element will consist of a pattern specified by discrete coordinates (e.g. a mask) relative to an origin. By choosing the coordinates, specific shapes can be formed (e.g. box, circle, hexagon, line).

- **Erosion and dilation**
  Erosion and dilation are the basic operations of MM which are used in derivation of the higher level operations. The erosion operator removes the part of the foreground that the structuring element does not completely fit. On the other hand, the dilation operator extends the foreground to contain coordinates which the specified structuring element and foreground has a non-empty intersection.

  Suppose X denotes the set of coordinates x corresponding to the input image A, S is the set of coordinates defined by the structuring element, and Sx denotes the translation of S so that its origin is at x. Then the erosion of A by S is defined as the set of points x such that Sx is a subset of X [5]:

  $$A \ominus S = \{x : S_x \subseteq X\} \tag{1}$$

  The opposite (dual) of erosion is dilation which is defined as set of all points x such that the intersection of Sx with X is non-empty [5]:

  $$A \oplus S = \left\{x : \hat{S}_x \cap X \neq \emptyset\right\} \tag{2}$$

  The extension of morphological operations to grey scale images is done by perceiving the image as a topological surface. Binary set operations intersection and union are replaced by the minimum and maximum operations respectively. For a grey scale image (f), grey scale erosion and dilation operations can be defined as in [5], respectively.

  $$\epsilon_S(f) = \min_{s \in S} f(x+s) \tag{3}$$

  $$\delta_S(f) = \max_{s \in S} f(x+s) \tag{4}$$

- **Opening and closing**
  The combination of dilation and erosion operations produces two new operations: opening and closing which depends on the order which operation is applied first. Opening is an erosion followed by a dilation [5]:

  $$\gamma_S(f) = \delta_S(\epsilon_S(f)) \tag{5}$$

  Closing is the opposite (dual) of the opening:

  $$\phi_S(f) = \epsilon_S(\delta_S(f)) \tag{6}$$

- **Area opening and area closing**
  These are the opening and closing operations which define a locally adaptable structuring element based on an area attribute instead of providing a fixed width/shape structuring element [7]. The area

opening operation removes an object if its area is below a defined threshold λ. Area opening for binary images is defined as follows [7]:

$$\gamma^a_\lambda(X) = \{x \in X | Area(C_x(X)) \geq \lambda\} \tag{7}$$

where $C_x(X)$ the connected opening or simply connected component of X. Area closing is defined by duality [7]:

$$\phi^a_\lambda(X) = [\gamma^a_\lambda(X^c)]^c \tag{8}$$

where X^c denotes the complement of set X. Grey level extensions can be defined by the union of different area operations in consecutive thresholded sets [7]. In grey scale images area opening removes a plateau, whereas area closing fills a hole, if its area is below a defined threshold.

- **Granulometry**
These are useful tools for obtaining *a priori* information about the size of the objects in the image. Granulometric size distributions are computed via a family of openings which have increasing, anti-extensive, idempotence properties [8, 9].

- **Top-hats**
A top-hat is the arithmetic difference between an image and its opening or closing. Top-hats by opening (white top-hat, WTH) and by closing (black top-hat, BTH) are defined as below [5]:

$$WTH(f) = f - \gamma_B(f) \tag{9}$$

$$BTH(f) = \phi_B(f) - f \tag{10}$$

The white top-hat extracts the brighter content of the image which is removed by opening. On the contrary the black top-hat extracts the darker content which is removed by closing. The extension to white *area* top-hats and black *area* top-hats is performed by using area opening and closing operations respectively [7].

- **Reconstruction**
This can be easily understood with the concept of *markers*. Many MM operations are extended to operate by an additional set (image) which marks particular components in the input to provide selective or conditional behaviour for elementary operations. For example, a marker controlled dilation operation is defined as follows [10]:

$$\delta_g(f) = \delta(f) \wedge g \tag{11}$$

where the elementary dilation operation is applied to the marker image (f) and then its point-wise minimum is calculated with the mask image (g). Therefore, the mask image limits the dilation operation. Since, they are limited by the mask image operations can be iterated to reach stability which leads to the definition of the morphological reconstruction. The reconstruction by the dilation operation is defined by iteration of elementary dilations until stability [10]:

$$R^\delta_g(f) = \delta^{(i)}_g(f), \quad \text{where} \quad \delta^{(i)}_g(f) = \delta^{(i+1)}_g(f) \tag{12}$$

- **Regional minima and maxima**
A *regional maximum* ($RMAX_h$) in a given value (h) of an image (f) is the connected components of pixels that have neighbour pixel values strictly lower than h. Thus, it is different than the *local maximum* that is defined by a pixel p that neighbours have values which are lower or equal to $f(p)$. The regional maximum of all h values, in an image is referred as regional maxima can be computed with reconstruction by dilation as follows [10]:

$$RMAX(f) = f - R^\delta_f(f - 1) \tag{13}$$

In a similar way, the regional minimum ($RMIN_h$) in a given value (h) of an image (f) is the connected components of pixels that have neighbour pixel values strictly higher than h. The regional minima can be computed with reconstruction by erosion as follows [10]:

$$RMIN(f) = R_f^{\epsilon}(f + 1) - f \tag{14}$$

- **The watershed transformation**
  The watershed transformation is a powerful morphological image segmentation tool. In a topographic representation of an image, the grey level value of each pixel can be perceived as an elevation at this point [11]. The watershed transformation, in an analogy to flooding simulations, tends to create partitions of the image corresponding to *catchment basins*. When the objects in the image correspond to minima, and the object boundaries are presented, the output of the transform divides the image plane into unique regions (influence zones of minima) associated with the objects.

Pattern classification

Pattern classification is a field in the broader paradigm, *Machine Learning*, which is concerned with learning of the computers. Definitions of some of the commonly used terms are provided below. More information can be found in various sources, e.g. [1, 12–14].

- **Feature**
  A feature is an observation or measurement which characterise and discriminate the different entities (e.g. objects, faces, sound, parasites) of the problem. A feature vector (also called pattern vector) may be comprised of many individual *features*. A process or function that analyses the image of the object and produce a feature vector is a *feature extractor*. An example of a feature to be used for pattern recognition can be a measurement of the binary objects such as area, width, and aspect ratio. However, a good feature must be robust against transformations and deformations of the image and colour/illumination variations.

- **Classifier**
  Classifier is a function which assigns a given feature vector associated with an observation to one of the classes that are defined in the problem. The simplest classifier can be a rule-based or heuristic-based function which performs the decision based on an if-then logic. However, in practice the problems are more complicated for heuristic decisions. Thus the most classifiers are designed to perform decisions based on more complex functions which are statistically justified on a set of observations (*training samples*).

- **Training samples and learning**
  More complex problems require increased number of features which result in higher dimensional *feature spaces*. Therefore classifier decisions require complex evaluations. In general, learning is an operation to reduce the classification error with an algorithm based on a mathematical model which refines the decision function. This is often performed using a set of pre-labelled (supervised) examples (*training samples*) which the decision function can be optimized on. The optimisation of the decision function with a labelled set of examples is often referred as *supervised learning*. In some problems the label information (supervision) for the training samples is not provided, which can be approached by *unsupervised learning* (or clustering). If the feature space is metric the classifier defines a decision boundary in the feature space, which is a partition for the classes. In simpler problems the decision boundary can be a linear hyperplane, however in practice most problems require non-linear and more complex decision surfaces.

- **Generalisation**
  By learning, a classifier aims to improve a decision capability for previously unseen samples. This is

known as *generalisation.* The learning process using training samples tends to reduce the classification error on training samples.

References

1. Davies R: *Machine Vision: theory, algorithms, practicalities 2nd Ed.* London, UK: Academic Press 1997.
2. Gonzalez RC, Woods RE: *Digital Image Processing.* Pearson Education, 3rd edition 2008.
3. Serra J: *Image Analysis and Mathematical Morphology.* Philadelphia, USA: Academic Press Inc 1983.
4. Dougherty ER, Lotufo RA: *Hands-on Morphological Image Processing.* USA: SPIE Press 2003.
5. Soille P: *Morphological Image Analysis.* Heidelberg, Germany: Springer-Verlag 2003.
6. Tek FB, Dempster AG, Kale I: **Noise sensitivity of watershed segmentation for different connectivity: experimental study**. *IEE Short Lett* 2004, **40**:1332–1333.
7. Vincent L: **Morphological area openings and closings for greyscale images**. In *Proc NATO Shape in Picture Workshop*, Driebergen, The Netherlands 1992.
8. Vincent L: **Granulometries and opening trees**. *Fundamenta Informaticae* 2000, **41**:57–90.
9. Maragos P: **Pattern spectrum and multiscale shape respresentation**. *IEEE Trans Pattern Anal Mach Intell* 1989, **2**:701–716.
10. Vincent L: **Morphological grayscale reconstruction in image analysis applications and efficient algorithms**. *IEEE Trans Image Process* 1993, **2**:176–201.
11. Vincent L, Soille P: **Watersheds in digital spaces: an efficient algorithm based on immersion simulations**. *IEEE Trans Pattern Anal Mach Intell* 1991, **13**:583–598.
12. Webb A: *Statistical pattern recognition 2nd Ed.* New York, USA: J Wiley and Sons Inc. 2002.
13. Bishop CM: *Neural Networks for Pattern Recognition.* UK: Oxford University Press 2004.
14. Duda R, Hart P, Stork D: *Pattern Classification.* New York, USA: Wiley-Interscience Publication 2000.
