# Supplementary material for: Computer vision for microscopy diagnosis of malaria
Source: Malar J. 2009 Jul 13;8:153. doi: 10.1186/1475-2875-8-153 (PMC2719653; doi:10.1186/1475-2875-8-153)
Supplement: Additional file 2 — Description of the performance measures. This file provides explanations of the accuracy related terms. [file 1475-2875-8-153-S2.pdf]

Description of performance measures

Independent of the test result (classification) a test sample can be *positive* (infected) or *negative* (not infected), as determined by another (reference) reliable method. There can be four different outcomes after the classification: (tp) true positive (the classification result is positive for a positive sample); (tn) true negative (the classification result is negative for a negative sample); (fp) false positive (the classification result is positive for a negative sample); (fn) false negative (the classification result is negative for a positive sample). The number of occurrences of these conditions can be used to analyse the diagnosis performance: TP, TN, FP, FN for tp, tn, fp, fn, respectively.

- **Sensitivity** (SE) is the proportion of the samples that are classified as positive among all the positive samples.

$$SE = \frac{TP}{TP + FN} \tag{1}$$

which is usually called the true detection rate (in the pattern recognition community) for binary pattern detection tasks. The higher the sensitivity, the more likely an infected person to be diagnosed as positive (i.e. sick).

- **Specificity** (SP) is the proportion of the samples that are classified as negative among all the negative samples. It is the probability of a negative result for a negative object (regular blood component, e.g. RBC, WBC, platelet, and artefact).

$$SP = \frac{TN}{TN + FP} \tag{2}$$

The higher the specificity, the less likely that a healthy blood component will be classified as a parasite. Consequently, a healthy person is more likely to be diagnosed as healthy. Sensitivity and specificity values of a diagnosis test should be interpreted together. In theory, for an ideal diagnosis method the values are independent and both can be as high as 1.0. It should be noted that assigning all the queries to one of the classes (consider the positive class) can simply achieve ($SE = 1.0$) but the specificity in this case would be $SP = 0.0$ which is not desirable.

In general pattern detection terms, the value $(1 - SP)$ is usually called the false detection rate.

- **Positive Prediction Value** is the proportion of the positive samples of all that are classified as positive.

$$PPV = \frac{TP}{TP + FP} \tag{3}$$

Positive prediction value indicates the reliability of a positive result. It answers a very intuitive question: what is probability of the patient being sick given the diagnosis is positive.

- **Negative Prediction Value** is the proportion of the negative samples of all that are classified as negative.

$$NPV = \frac{TN}{TN + FN} \tag{4}$$

Negative prediction value is the opposite of PPV. It answers the question: what is the probability of the patient being healthy given the diagnosis is negative.

Sensitivity and specificity values are not affected by the number of positive and negative samples among the test set (i.e. sample distribution). However, prediction values are sensitive to the sample distribution (see [1] for a broader explanation of the terms).

References

1. Loong TW: **Understanding sensitivity and specificity with the right side of the brain**. *BMJ* 2003, **327**:716–719.
